# Combinations of reproductive, individual, and weather effects best explain torpor patterns among female little brown bats (*Myotis lucifugus*)

**DOI:** 10.1002/ece3.5091

**Published:** 2019-04-16

**Authors:** Nicole K. Besler, Hugh G. Broders

**Affiliations:** ^1^ Department of Biology Saint Mary's University Halifax Nova Scotia Canada; ^2^ Department of Biology University of Waterloo Waterloo Ontario Canada

**Keywords:** Chiroptera, ecophysiology, *Myotis lucifugus*, reproduction, thermoregulation, torpor

## Abstract

Heterothermic mammals can use torpor, a state of metabolic suppression, to conserve energy during times of limited food and poor environmental conditions. Females may use torpor throughout gestation and lactation; however, there are associated physiological and ecological costs with potential fitness consequences. Previous studies have controlled for, but not quantified the impact of interindividual variation on torpor patterns and understanding this may provide insight on why certain thermoregulatory responses are employed. The objective of this study was to identify and quantitatively characterize the intrinsic variables and weather conditions that best explain variation in torpor patterns among individual female little brown bats, *Myotis lucifugus*. We used temperature‐sensitive radio‐transmitters affixed to females to measure skin temperature patterns of 35 individuals roosting in bat boxes in the spring and summer. We used Bayesian multi‐model inference to rank a priori‐selected models and variables based on their explanatory power. Reproductive condition and interindividual effects best explained torpor duration and depth, and weather best explained torpor frequency. Of the reproductive conditions, lactating females used torpor for the shortest durations and at shallower depths (i.e., smallest drop in minimum *T*_sk_), while females in early spring (i.e., not‐obviously‐pregnant) used torpor for the longest and deepest. Among individuals, the greatest difference in effects on duration occurred between pregnant individuals, suggesting interindividual variation within reproductive condition. Increases in precipitation and wind were associated with a higher probability of torpor use. Our results provide further support that multiple variables explain torpor patterns and highlight the importance of including individual effects when studying thermoregulatory patterns in heterothermic species.

**OPEN RESEARCH BADGES:**

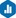

This article has earned an Open Data Badge for making publicly available the digitally‐shareable data necessary to reproduce the reported results. The data is available at https://doi.org/10.5061/dryad.c04tj85.

## 1 INTRODUCTION

Thermoregulatory responses are hypothesized to have evolved to help individuals sustain physiological function and activity (e.g., foraging) necessary for survival and reproduction (Dowd, King, & Denny, 2015; Scholander, Hock, Walters, & Irving, 1950). Mammals and birds typically have high metabolic rates to maintain an elevated body temperature (*T*_b_), which allows sustained foraging and reproduction in a range of environmental conditions (e.g., cold ambient temperatures [*T*_a_]) (Heinrich, 1977; Scholander et al., 1950). However, a high metabolic rate is energetically expensive, especially for small‐bodied animals that lose heat more rapidly to the environment than larger‐bodied animals (Aschoff, 1981). Further, reproduction increases energetic demands, particularly for female mammals as pregnancy and lactation require a substantial amount of energy (Gittleman & Thompson, 1988; Racey, Speakman, & Swift, 1987).

Some small‐bodied mammals and birds are classified as heterotherms and can use torpor, a state of metabolic suppression and reduced *T*_b _(Geiser, 2004; Wang & Wolowyk, 1988), to conserve energy during times of inactivity, limited food sources, and cold or hot and arid conditions (Smit, Harding, Hockey, & Mckechnie, 2013; Wang & Wolowyk, 1988; Wojciechowski, Jefimow, & Tegowska, 2007). Torpor may be used to survive severe and unpredictable weather conditions, for example, sugar gliders increased torpor use during a severe storm, potentially to compensate for lost foraging opportunities (Nowack, Rojas, Körtner, & Geiser, 2015). However, torpor slows physiological processes, including those involved in reproduction (e.g., incubation, fetal development, milk production) and females must balance the immediate energetic advantages of torpor use with the costs on their reproductive output (Calder & Booser, 1973; Racey & Swift, 1981; Wilde, Knight, & Racey, 1999). As such, the patterns and extent of torpor use by reproductive females vary among taxa and environmental conditions (McAllan & Geiser, 2014). Torpor use during incubation, brooding, pregnancy, or lactation occurs among species of hummingbirds, marsupials, bats, tenrecs, hedgehogs, mouse lemurs, and dormice (Calder & Booser, 1973; Dausmann, 2014; Dzal & Brigham, 2013; Fowler, 1988; Geiser et al., 2008; Juškaitis, 2005; Lovegrove & Génin, 2008). These species typically inhabit unpredictable environments where the availability of food and water fluctuates with season or weather, in which torpor use may be necessary for reproduction to occur or to optimize reproductive output (Körtner, Pavey, & Geiser, 2008; McAllan & Geiser, 2014). In some species, torpor is only observed during parts of reproduction, such as early pregnancy (Fowler, 1988; Körtner et al., 2008), while in others it is observed in both pregnancy and lactation (Dzal & Brigham, 2013; Geiser et al., 2008). Further, under captive conditions, neither sex nor reproductive condition affected torpor use, which suggests that ecological, rather than physiological, factors determine the costs and benefits of thermoregulatory responses during reproduction (Turbill & Geiser, 2006).

Insectivorous bats in temperate regions (Chiroptera: Vespertilionidae) are unique model organisms for understanding how torpor can be used for survival and reproduction. These species have high energetic demands due to their small size (typically 5–35 g) (Aschoff, 1981), reliance on fluctuating food sources (i.e., insects) (Anthony, Stack, & Kunz, 1981), and flight being energetically expensive (Thomas & Suthers, 1972). Temperate bats are among the few taxa that are known to use torpor during both pregnancy and lactation in the wild and may need to employ various thermoregulatory responses to maximize fitness (Audet & Fenton, 1988; Dzal & Brigham, 2013; Hamilton & Barclay, 1994; Lausen & Barclay, 2003; Rintoul & Brigham, 2014). Torpor patterns expressed among female bats in different reproductive and weather conditions have varied inter‐and intraspecifically and geographically (Dzal & Brigham, 2013; Johnson & Lacki, 2014; Lausen & Barclay, 2003; Rintoul & Brigham, 2014; Solick & Barclay, 2007). Interindividual variation within reproductive condition may provide an additional, previously unquantified, explanation for this discrepancy.

Variation in torpor use by female bats in each reproductive condition may be explained by the costs and benefits to fitness. In some study systems, pregnant females used torpor less frequently, at shallower depths, and for shorter durations than lactating females (Dzal & Brigham, 2013; Studier & O'Farrell, 1972). This suggests that, in some cases, the high energetic demands of lactation necessitates greater torpor use and that the cost of delaying parturition is greater than delaying weaning (Dzal & Brigham, 2013; Studier & O'Farrell, 1972). However, in other study systems, lactating females used torpor at shallower depths than pregnant females suggesting that the cost of deep torpor is greater on juvenile growth than fetal development (Chruszcz & Barclay, 2002; Lausen & Barclay, 2003). In some cases, reproductive condition did not influence torpor frequency (Chruszcz & Barclay, 2002; Johnson & Lacki, 2014; Rintoul & Brigham, 2014). The variation in torpor patterns among reproductive conditions suggests that individual and environmental factors may influence how torpor is used during each reproductive condition (Chruszcz & Barclay, 2002; Dzal & Brigham, 2013; Johnson & Lacki, 2014).

Interindividual variation in energy balance may influence thermoregulatory responses. For example, genetically determined differences in basal metabolic rates and behavior (e.g., boldness and aggression) influence energetic expenditure and intake (Biro & Stamps, 2008; White & Kearney, 2013). The resulting energy balance influences body condition, which may affect torpor use (Rambaldini & Brigham, 2008; Vuarin, Dammhahn, & Henry, 2013). In some cases, torpor is used in response to energetic constraints imposed by having a low body mass (Rambaldini & Brigham, 2008). However, rewarming to normal *T*_b_ via endogenous heat production will require energy; therefore, individuals in poor body condition may be limited in torpor use (Vuarin et al., 2013). Differences in energy acquisition may result in some individuals employing different thermoregulatory strategies in response to environmental fluctuations (Hickey & Fenton, 1996) and may have different consequences for reproductive success (Dammhahn, Landry‐Cuerrier, Reale, Garant, & Humphries, 2016).

Weather conditions may increase energetic demands for reproductive female bats in regions with cool climates (Klug‐Baerwald, Gower, Lausen, & Brigham, 2016; Racey et al., 1987; Scholander et al., 1950; Voigt, Schneeberger, Voigt‐heucke, & Lewanzik, 2011). Low *T*_a_, wind, and precipitation increase heat loss (Klug‐Baerwald et al., 2016; Scholander et al., 1950; Voigt et al., 2011) and reduce insect (i.e., prey) activity (Anthony et al., 1981; Racey et al., 1987) and, therefore, the potential for energy acquisition. Some authors found weather to be an important predictor for the duration and depth of torpor in temperate bats (Dzal & Brigham, 2013; Johnson & Lacki, 2014; Klug & Barclay, 2013), while in other study systems variation in *T*_a_ did not affect torpor patterns (Rintoul & Brigham, 2014). These inconsistencies may be due to weather conditions varying among reproductive stages (Chruszcz & Barclay, 2002) and, in some cases, torpor use may be necessary to survive poor spring conditions and optimize reproductive timing (Willis, Brigham, & Geiser, 2006). For example, in regions with cold spring weather, torpor use during pregnancy may be advantageous as females can conserve energy and time parturition to coincide with the most favorable environmental conditions for offspring survival (i.e., higher insect availability and warmer temperatures) (Willis et al., 2006). Thus, weather may influence the costs and benefits of torpor use (Chruszcz & Barclay, 2002; Willis et al., 2006) and the effects may be multiplicative (Klug & Barclay, 2013; Klug‐Baerwald et al., 2016).

**Figure 1:**
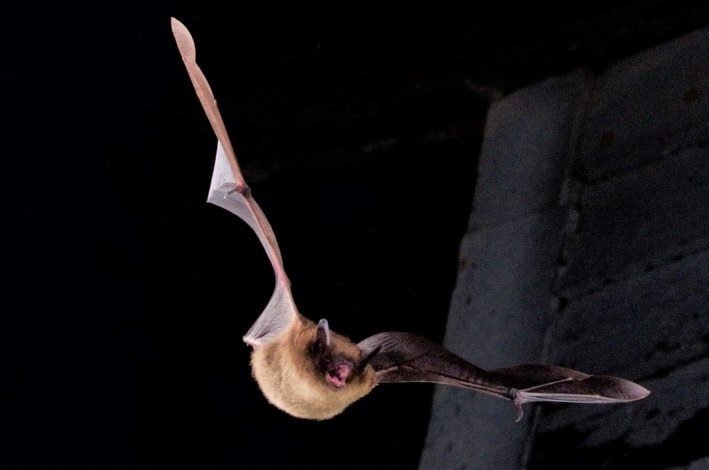
Little brown bat, *Myotis lucifugus*, flying out of a bat box at Salmonier Nature Park, Newfoundland, Canada. Photo credit: Cody Fouts

Little brown bats (*Myotis lucifugus*) are small, insectivorous bats found across North America (Fenton & Barclay, 1980; Figure 1) and use torpor during pregnancy and lactation (Dzal & Brigham, 2013). Newfoundland is on the eastern edge of the range of *M. lucifugus* (Fenton & Barclay, 1980) and the cold, wet climate (Banfield, 1983) makes it a good study system for understanding thermoregulatory responses to energetic constraints from environmental conditions. In south‐eastern Newfoundland, where the study took place, the daily average summer temperature is 14°C, annual precipitation is 1,200–1,700 mm, and the average wind speed is 20 km/hr (Banfield, 1983; Khan & Iqbal, 2004). Additionally, the last frost day typically occurs in mid to late May (Banfield, 1983), resulting in a delay to the onset of warm temperatures and likely insect abundance (Anthony et al., 1981; Racey et al., 1987). The objective of our study was to quantitatively characterize intrinsic variables and weather conditions that explain variation in torpor use among female *M. lucifugus*. Most studies evaluated the effects of reproductive condition and weather while controlling for individual when evaluating torpor patterns in temperate bat species (Dzal & Brigham, 2013; Johnson & Lacki, 2014; Rintoul & Brigham, 2014); however, individual effects may be important. Therefore, we hypothesized that (1) reproductive condition, (2) interindividual variation, and (3) weather would explain variation in torpor frequency, duration, and depth (i.e., minimum skin temperature [*T*_sk_]). These metrics represent different extents of torpor use with energetic savings and physiological consequences increasing at varying degrees as frequency, duration, and depth increase (Willis & Brigham, 2003). Given that torpor use delays parturition and weaning (Racey & Swift, 1981; Wilde et al., 1999), we predicted that pregnant and lactating females would use torpor less frequently, for shorter durations, and shallower depths than post‐lactating and nonreproductive females. We expected pregnant females and females in early spring (i.e., not‐obviously‐pregnant) to use torpor more frequently, for longer durations, and greater depths than lactating females as delaying parturition would be less costly than delaying weaning in Newfoundland given the cool and wet spring conditions. Given that physiological variation exists among individuals (e.g., metabolic rate) and likely affects energy acquisition (Biro & Stamps, 2008; White & Kearney, 2013), we predicted interindividual variation in torpor patterns under the same reproductive conditions. Finally, we predicted that low *T*_a_, high wind speed, and precipitation would be associated with increased torpor frequency, duration, and depth (i.e., lower minimum *T*_sk_) due to environmental conditions affecting energetic demands (Racey et al., 1987; Scholander et al., 1950; Voigt et al., 2011).

## 2 METHODS

### 2.1 Data collection

Thermoregulatory data were collected from June to August 2016–2017 from female *M. lucifugus *roosting in bat boxes at Salmonier Nature Park, on the Avalon Peninsula in south‐eastern Newfoundland (47°15′53.28″N, 53°17′2.04″W). Mist nets (Avinet Inc., Dryden, NY, USA) were used to capture bats and upon capture, individuals were assigned to a reproductive class. Pregnancy was determined by gently palpating the abdomen to detect a fetus. Bats were considered lactating if bare patches around the nipples were present, and milk could be expressed and post‐lactating if there was hair regrowth around the nipples and milk could not be expressed (Racey, 1988; Racey & Swift, 1981). Capture data were used to estimate the timing of parturition and juvenile volancy, which was determined as the difference between the first lactating female caught and the first volant juvenile caught. Precalibrated temperature‐sensitive radio‐transmitters (Pip31; Lotek Wireless Inc., Newmarket, ON, Canada), weighing 0.37 g, were used to measure *T*_sk_ as an indicator of *T*_b_ (Barclay et al., 1996). Transmitters were attached between the scapulae of females using surgical cement (Torbot Group Inc., Cranston, Rhode Island, USA) after trimming the fur. Additionally, a passive integrated transponder tag (Trovan Micro Transponder; Dorset Group, Aalten, The Netherlands) was inserted sub‐dermally between the scapulae for individual identification (Burns & Broders, 2015). All handling procedures were done in accordance with the Canadian Council on Animal Care, approved by Saint Mary's University Animal Care Committee and under a permit from the province of Newfoundland and Labrador (permit # WLR2016‐12 and WLR2017‐16).

*T*_a_ was recorded every 10 min within the study site using temperature and humidity data loggers (±0.5°C, Hygrochron iButton, DS1923; Embedded Data Systems, Lawrenceburg, KY, USA) placed in the shade, 2 m above ground. Daily maximum wind speed (km/hr) and total precipitation (mm) data were taken from the nearest weather station that logs data (St. John's; 47°37′ N, 52°45′ W; Environment Canada 2016, 2017; 57 km from Salmonier Nature Park). In 2017, maximum wind speed (km/hr) and total precipitation (mm) data were recorded daily from a weather station closer to the study site (Brigus Junction; 47°26′ N, 53°33′ W; Weather Underground, 2017; 26 km from Salmonier Nature Park). *T*_sk_ data were collected each day a transmitter was active or until it fell off. Data logging receivers (SRX800‐D1 and SRX400; Lotek Wireless Inc.) and 3‐ and 5‐element Yagi antennas were placed below bat boxes to record the interpulse intervals of the transmitters every 10 min. The interpulse intervals of the transmitters were converted to temperature based on the transmitter‐specific calibration curve. When logging receivers did not log the interpulse interval of a transmitter, a voice recorder (HTC One M8; HTC, New Taipei City, Taiwan) was used to record the transmitter beeps from a manual receiver (SRX800; Lotek Wireless Inc.). The number of beeps per minute was manually counted from the recordings every 10 min.

### 2.2 Defining torpor

Current methods for defining a torpor threshold based on *T*_sk_ are biased for at least some individuals as transmitter readings of *T*_sk_ may result in differences from *T*_b _up to 6°C (McKechnie, Ashdown, Christian, & Brigham, 2007; Willis, 2007). Additionally, metabolically determined thresholds cannot be inferred from *T*_sk_ without *T*_b _measurements (Willis, 2007). Some individuals in our study maintained low *T*_sk_ (i.e., <32°C) for prolonged periods and a threshold based on the active temperature (Barclay, Lausen, & Hollis, 2001) or the modal method (McKechnie et al., 2007) may underestimate torpor use. We defined a threshold as 3°C less than the 80th percentile of all *T*_sk_ for each individual as this yielded the most reasonable threshold for all individuals. A bat was classified as torpid anytime *T*_sk _fell below the torpor threshold (*T*_onset_) for ≥2 consecutive readings (20 min). A bat day was defined as the final time a bat arrived at a roost until it emerged the following night. If a bat did not leave the roost overnight, then a bat day was defined as starting at midnight and ending at 23:50 on the same day. Bat days missing >60 min of *T*_sk_ data were not used in the analysis. Torpor frequency was defined as the proportion of bat days for which *T*_sk_ dropped below the threshold. Torpor duration was measured as the total number of minutes per day, from all torpor bouts, for which *T*_sk_ was below *T*_onset_ and torpor depth was defined as the minimum *T*_sk_ (°C) recorded in one bat day.

### 2.3 Statistical analysis

We conducted multi‐model inference (Burnham & Anderson, 2002) using a Bayesian approach to determine which combinations of reproductive condition, individual, and weather best explain variation in torpor patterns (frequency, duration, and depth). Bayesian inference estimates the probability of a model parameter being a certain value given the data and allows for individual effects to be quantified (Kruschke, 2015; Kruschke & Liddell, 2018). Bayesian hierarchical modeling provides descriptive parameters for each individual and accounts for individuals with multiple data points (nonindependence) (Kruschke, 2015; Kruschke & Liddell, 2018). We used Markov chain Monte Carlo (MCMC) methods to randomly sample parameter values from a probability distribution to approximate the posterior estimate distribution of a parameter (Kruschke, 2015). The mean value of the resulting posterior distribution indicates the most likely estimate and represents the amount of deflection above or below the mean value of *y* across all groups for all predictor variables (*β*_0_) (Kruschke, 2015). Uncertainty in parameter estimates is indicated by the span of the 95% highest density interval (HDI), where values within the interval have a higher probability density than points outside the interval (Kruschke, 2015; Kruschke & Liddell, 2018). Our study used weakly informed prior distributions that came from a normal distribution with a mean (*μ*) of 0 and a high standard deviation, thereby assuming equal probabilities across possible values (Kruschke, 2015).

We generated a set of 14 a priori models that included predictor variables (reproductive condition, individual, and weather variables) on their own and in combinations. A logistic regression was used for torpor frequency (torpid/not torpid) models with the global model having the following equation:

$$\mu_{i}=\text{ilogit}\left(\beta_{0}+\sum_{j}\beta_{1}x_{1[i,j]}+\beta_{2}\left(x_{2[j]}-\overset{-}{x}\right)+\sum_{k}\beta_{3}x_{3[i,k]}+\beta_{4}\left(x_{4[j]}-\overset{-}{x}\right)+\beta_{4}\left(x_{4[j]}-\overset{-}{x}\right)\right).$$

The models for torpor duration (minutes) and depth (°C) were run as multiple linear regressions with the global model having the following equation:

$$\mu_{i}=\beta_{0}+\sum_{j}\beta_{1}x_{1[i,j]}+\beta_{2}\left(x_{2[j]}-\overset{-}{x}\right)+\sum_{k}\beta_{3}x_{3[i,k]}+\beta_{4}\left(x_{4[j]}-\overset{-}{x}\right)+\beta_{4}\left(x_{4[j]}-\overset{-}{x}\right).$$

The predicted values (*μ*_i_) for torpor frequency came from a Bernoulli distribution, and the values for torpor duration and depth came from a normal distribution. Repeated measures were taken from most individuals; therefore, the models containing reproductive condition and individual were made hierarchical. Each reproductive condition came from a normal distribution containing individuals, and each individual came from a normal distribution containing days.

We ranked models using the deviance information criterion (DIC), with the lowest DIC value indicating the most explanatory model (Spiegelhalter, Best, Carlin, & Linde, 2002). DIC is based on the posterior distribution of the deviance (log‐likelihood) and is a useful model selection criterion for hierarchical Bayesian models where the posterior distributions are obtained from MCMC methods (Spiegelhalter et al., 2002). For each model, we took the difference between the DIC value and that of the best model (∆*i*). Using ∆*i*, we calculated the DIC weights (*w_i_*), the likelihood that the *i*th model is the best model, for each candidate model using the same equation as Akaike weights (Burnham & Anderson, 2002). We then calculated the sum of the weights (∑*w_i_*) for the *i*th model up to the highest ranking model for models constituting ≥95% of the weights (Burnham & Anderson, 2002). Not all variables within models in the 95% confidence set may be explanatory; therefore, we calculated the normalized weight for each variable (Burnham & Anderson, 2002). A normalized variable weight (N*w_i_*) >0.60, arbitrarily selected, indicated explanatory effects (e.g., Garroway & Broders, 2005). For variables with N*w_i_* > 0.60, we calculated the model‐averaged posterior estimates, unconditional standard deviations (Burnham & Anderson, 2002), and the model‐averaged *β*_0_. For torpor frequency, the model‐averaged posterior estimates were used to determine the odds ratio (exp[posterior estimate × unit of increase]) (Hosmer & Lemeshow, 2013). All statistical analyses were conducted in R version 3.4.2 (R Core Team, 2016). The R package runjags version 2.0.4‐2 was used to interface JAGS version 4.2.0 (Denwood & Plummer, 2016).

## 3 RESULTS

### 3.1 Sample characteristics

**Figure 2:**
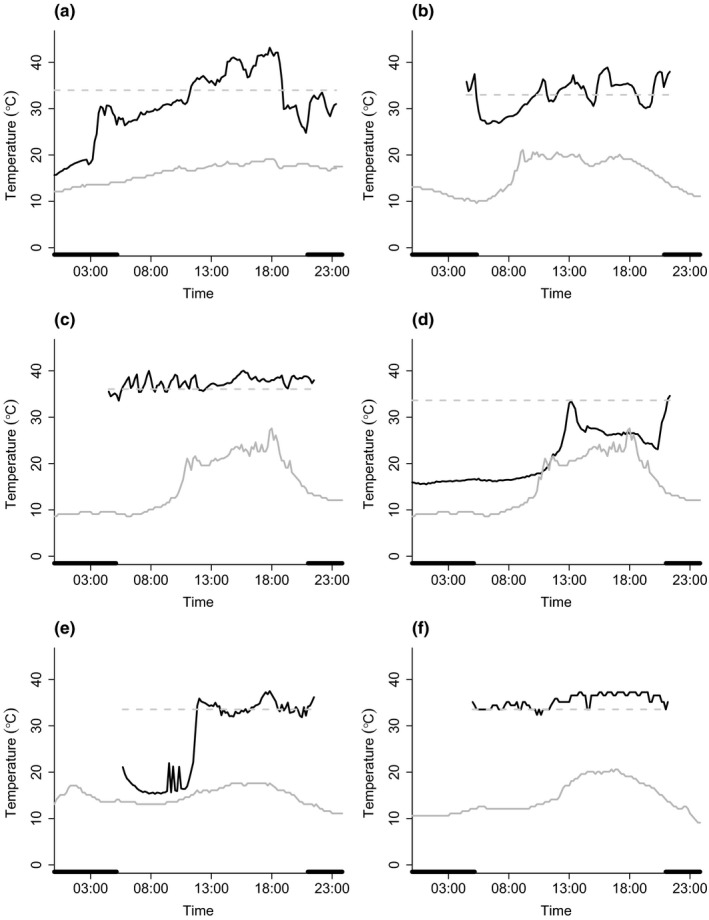
Skin temperature (*T*_sk_) patterns of the same individual female *Myotis lucifugus* during (a) pregnancy and (b) lactation, two pregnant individuals (c) #13 and (d) #17 on June 30, 2017, and the same pregnant individual on (e) July 3, 2017 and (f) July 4, 2017. Data were collected from bats roosting in bat boxes at Salmonier Nature Park, Newfoundland. The black line represents *T*_sk_ (°C), the dashed gray line represents the torpor onset threshold (°C), and the gray line represents ambient temperature (°C). Black bars above the *x* axis represent night

We collected thermoregulatory data from 11 pregnant, 11 lactating, eight post‐lactating, two nonreproductive, and four not‐obviously‐pregnant females over 153 bat days. Data from not‐obviously‐pregnant females were collected from June 16 to 20, 2016, and from June 12 to 26, 2017. Pregnant females were sampled between June 26 and July 8, 2017; however, data from 2016 were not used in the analysis due to incomplete data days from transmitter failure. Nonreproductive females were sampled between June 29 and July 4, 2016. Lactating females were sampled from July 18 to 24 and on August 12, 2016, and from July 17 to 28, 2017. Data on post‐lactating females were collected from August 2 to 4, 2016, and August 4 to 16, 2017. The mean ± *SD* temperature when each reproductive condition was sampled varied and was 12.1 ± 2.7°C for not‐obviously‐pregnant females, 13.6 ± 2.6°C for pregnant females, 16.3 ± 2.5°C for nonreproductive females, 17.0 ± 1.8°C for lactating females, and 16.7 ± 2.0°C for post‐lactating females. Additionally, one individual was captured as pregnant and tracked again during lactation. This individual exhibited longer torpor bouts at greater depths (i.e., lower minimum *T*_sk_) during pregnancy (950.0 min/day; 15.7°C) than lactation (480.0 min/day; 26.7°C) on days where mean *T*_a_ was similar (16.3 and 15.7°C; Figure 2a,b). Thermoregulatory patterns also varied among individuals in the same reproductive condition on the same day (Figure 2c,d), and among days for the same individual (Figure 2e,f). The first lactating females caught were on July 16 in 2016 and July 14 in 2017 and the first volant juveniles caught were July 29 in 2016 and July 28 in 2017. The greatest mean *T*_a_ during our study occurred on July 17 for 2016 (24.3°C) and 2017 (19.9°C), shortly after the dates of the approximated first parturition.

### 3.2 Model selection

Each torpor pattern was best explained by different models and predictor variables (Tables 1 and 2). The model that best explained variation in torpor duration (*w_i_* = 0.48) and depth (*w_i_* = 0.91) was a bivariate model that included reproductive condition and individual effects. The model that best explained torpor frequency (*w_i_* = 0.33) contained the weather variables maximum wind speed and precipitation. Maximum wind speed was an additional variable in the highest ranked model for torpor depth. Reproductive condition occurred in three of the five models within the 95% confidence set for torpor duration and all models for depth and had a normalized variable weight of 0.85 and 1.00, respectively. Individual effects occurred in all models within the 95% confidence set for torpor duration and depth. Precipitation occurred in the top two models within the 95% confidence set for torpor frequency and had a normalized variable weight of 0.81. Maximum wind speed occurred in five of the ten models within the 95% confidence set for torpor frequency and one of the two models for depth and had a normalized variable weight of 0.62 and 0.93, respectively.

**Table 1:** Deviance information criterion (DIC) values, difference between DIC values of the *i*th model and the highest ranked model (∆*i*), DIC weights (*w_i_*), and the sum of the DIC weights of the *i*th model and all higher‐ranking models constituting ≥95% of the DIC weights (∑*w_i_*) for models explaining variation in torpor frequency, duration, and depth among female *Myotis lucifugus* roosting in bat boxes in Newfoundland, Canada from June to August 2016 and 2017

Model	DIC	∆*i*	*w_i_*	*∑w_i_*
Frequency				
Precipitation + Wind	89.12	0.00	0.33	0.33
Precipitation	89.88	0.75	0.23	0.56
Precipitation + Wind + Reproductive + Min *T*_a_	91.64	2.52	0.09	0.66
Wind	92.33	3.21	0.07	0.72
Precipitation + Wind + Min *T*_a_ + Individual	92.35	3.23	0.07	0.79
Precipitation + Reproductive + Individual	92.67	3.54	0.06	0.85
Wind + Min *T*_a_	93.71	4.58	0.03	0.88
Min *T*_a_	93.92	4.80	0.03	0.91
Individual	94.21	5.09	0.03	0.94
Reproductive	94.46	5.34	0.02	0.96
Duration				
Reproductive + Individual	343.10	0.00	0.48	0.48
Reproductive + Individual + Min *T*_a_	344.94	1.83	0.19	0.68
Reproductive + Individual + Precipitation	345.44	2.33	0.15	0.83
Individual + Min *T*_a_ + Precipitation + Wind	346.02	2.92	0.11	0.94
Individual	348.19	5.08	0.04	0.98
Depth				
Reproductive + Individual + Wind	322.76	0.00	0.91	0.91
Reproductive + Individual	325.04	2.28	0.07	0.97

Models were ranked based on DIC values, with lowest value explaining more of the variation in the data.

Min *T*_a_: minimum ambient temperature.

**Table 2:** Normalized weights for variables (N*w_i_*) in models constituting ≥95% of the deviance information criterion weights to explain variation in torpor frequency, duration, and depth

Torpor characteristic	Reproductive	Individual	Min *T*_a_	Wind	Precipitation
Frequency	0.18	0.16	0.23	**0.62**	**0.81**
Duration	**0.85**	**1.00**	0.31	0.11	0.27
Depth	**1.00**	**1.00**	0.00	**0.93**	0.00

Variables with N*w_i_* < 0.60 were not considered to have explanatory effects. Bolded values indicate variables that occurred in the highest ranked model.

Min *T*_a_: minimum ambient temperature.

### 3.3 Reproductive condition

**Figure 3:**
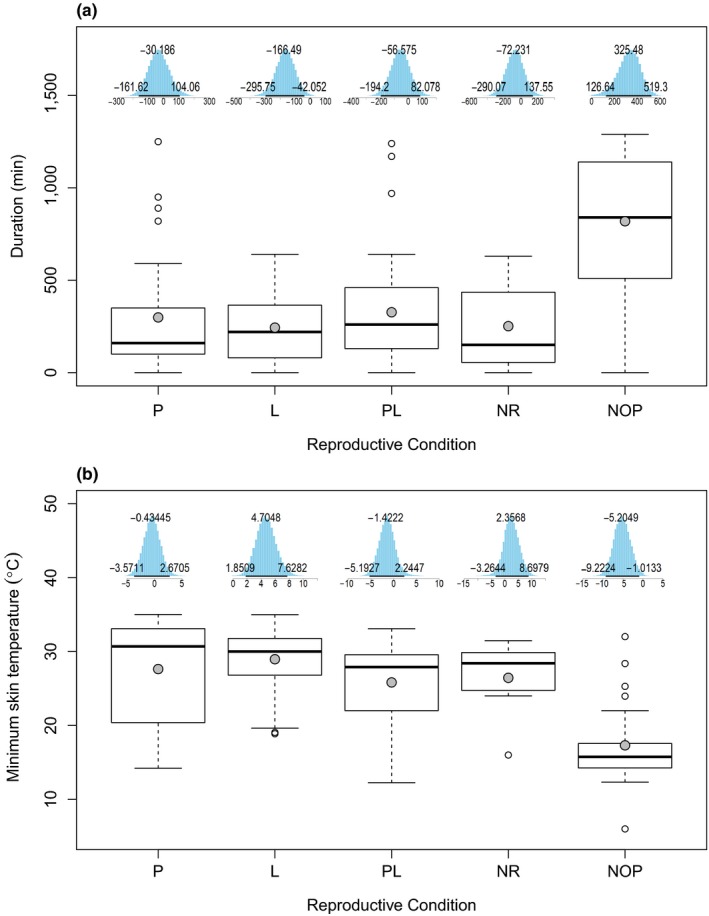
Difference in torpor (a) duration and (b) depth for pregnant (*n* = 11, *N* = 30), lactating (*n* = 11, *N* = 43), post‐lactating (*n* = 8, *N* = 37), nonreproductive (*n* = 2, *N* = 7), and not‐obviously pregnant (*n* = 4, *N* = 22) female *Myotis lucifugus* in Newfoundland from June to August 2016 and 2017. The top and bottom of each box show the upper and lower quartiles and the dashed vertical lines represent the maximum and minimum values. The black bars represent the median, the gray dots represent the mean, and open circles represent outliers. Above the boxes are the posterior distributions for the estimates of each reproductive condition from the highest ranked candidate model. L: lactating; *n*: number of individual bats; *N*: number of bat days; NOP: not‐obviously‐pregnant; NR: nonreproductive; P: pregnant, PL: post‐lactating

The mean ± *SD* torpor duration for pregnant (298.7 ± 318.7 min/day), lactating (243.5 ± 191.8 min/day), post‐lactating (326.8 ± 301.1 min/day), and nonreproductive (251.4 ± 247.1 min/day) females were lower than not‐obviously‐pregnant females (819.1 ± 387.7 min/day) (Figure 3a). The longest torpor bout recorded was 1, 290 min (21.5 hr) from a not‐obviously‐pregnant female. The model‐averaged mean torpor duration across all reproductive conditions (*β*_0_) was 377.5 min/day (Table 3). The not‐obviously‐pregnant condition was the only reproductive condition to result in an increase in torpor duration from *β*_0 _with the lactating condition resulting in the greatest decrease. Belonging to the not‐obviously‐pregnant condition most likely increased time spent torpid by 314.7 min/day above *β*_0_ (377.5 min/day), whereas being in the lactating condition most likely decreased duration by 155.8 min/day below *β*_0_. The model‐averaged posterior estimates were similar among the pregnant, post‐lactating, and nonreproductive conditions; however, the 95% HDI of the posterior distributions all overlapped zero, indicating uncertainty in the direction of the estimates (Figure 3a).

**Table 3:** The model‐averaged posterior estimates ($\hat{\theta }$), unconditional standard deviations (*SD*), 95% highest density interval (HDI) from the highest ranked model, and the model‐averaged *β*_0_ for variables with a normalized weight >0.60 for torpor frequency, duration, and depth

Variable	Posterior estimate ($\hat{\theta }$)	*SD*	95% HDI	*β*_0_
Frequency				
Wind	0.03	0.02	−0.01, 0.08	2.11
Precipitation	0.31	0.20	0.00, 0.74	1.78
Duration				
Pregnant	−36.50	69.51	−163.67, 102.39	377.49
Lactating	−155.75	67.52	−292.82, −44.06	377.49
Post‐lactating	−50.86	70.82	−191.33, 81.79	377.49
Nonreproductive	−71.60	108.09	−289.35, 136.10	377.49
Not‐obviously‐pregnant	314.71	105.16	123.31, 514.46	377.49
Individual #13	−152.62	103.80	−372.85, 30.75	370.31
Individual #17	208.16	165.52	−72.41, 533.47	370.31
Depth				
Pregnant	−0.38	1.58	−3.57, 2.67	25.42
Lactating	4.71	1.47	1.85, 7.63	25.42
Post‐lactating	−1.34	1.89	−5.19, 2.24	25.42
Nonreproductive	2.22	3.03	−3.26, 8.70	25.42
Not‐obviously‐pregnant	−5.20	2.09	−9.22, −1.01	25.42
Individual #13	6.04	2.16	1.78, 10.19	25.42
Individual #12	−4.43	2.47	−9.40, 0.31	25.42
Wind	−0.09	0.08	−0.24, 0.03	25.45

Only the posterior estimates for the individual with the most negative value and for the individual with the most positive value for duration and depth are displayed for brevity.

The mean ± *SD* minimum *T*_sk_ for pregnant (27.6 ± 8.2°C), lactating (29.0 ± 4.2°C), post‐lactating (25.8 ± 5.7°C), and nonreproductive (26.4 ± 5.3°C) females were lower than not‐obviously‐pregnant females (17.3 ± 5.8°C) (Figure 3b). The lowest *T*_sk_ recorded was from a not‐obviously‐pregnant female and was 6.0°C. The model‐averaged mean minimum *T*_sk_ across all reproductive conditions (*β*_0_) was 25.4°C. The lactating and nonreproductive conditions resulted in an increase from *β*_0 _while the pregnant, post‐lactating, and not‐obviously‐pregnant conditions resulted in a decrease. The greatest difference occurred between the lactating and not‐obviously‐pregnant conditions where belonging to the lactating condition most likely increased minimum *T*_sk_ by 4.7°C above *β*_0_ (25.4°C) and belonging to the not‐obviously‐pregnant condition most likely decreased minimum *T*_sk_ by 5.2°C below *β*_0_.

### 3.4 Individual

The model‐averaged mean torpor duration and minimum *T*_sk_ across all individuals (*β*_0_) was 370.3 min/day and 25.4°C, respectively. The individuals that resulted in the greatest increase and decrease in torpor duration from *β*_0 _were both pregnant females and exhibited different thermoregulatory patterns on the same day (Figure 2c,d). Individual #13 most likely decreased time spent torpid by 152.6 min/day and individual #17 most likely increased time spent torpid by 208.2 min/day. The individual with the greatest increase in minimum *T*_sk_ was the same pregnant individual with the greatest decrease in torpor duration and most likely increased minimum *T*_sk_ by 6.0°C. The individual that resulted in the greatest decrease in minimum *T*_sk_ was a not‐obviously‐pregnant female and likely decreased minimum *T*_sk_ by 4.4°C.

### 3.5 Weather

**Figure 4:**
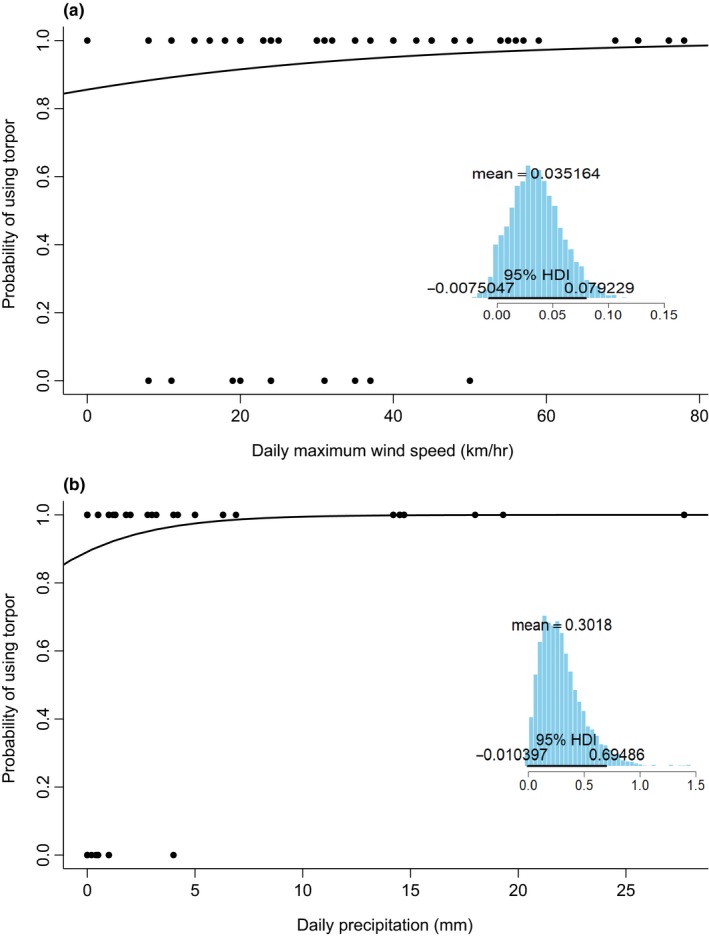
Torpor frequency (i.e., the probability of using torpor on a given day) for female *Myotis lucifugus* in Newfoundland as mean daily (a) maximum wind speed and (b) precipitation increase. Curves are logistic regressions (*y* = exp(*β*_0_ + *β*_1_)/(1 + exp(*β*_0_ + *β*_1_))) based on the model‐averaged posterior estimates. The posterior distribution of each variable for the highest ranked model is adjacent to the plot

**Figure 5:**
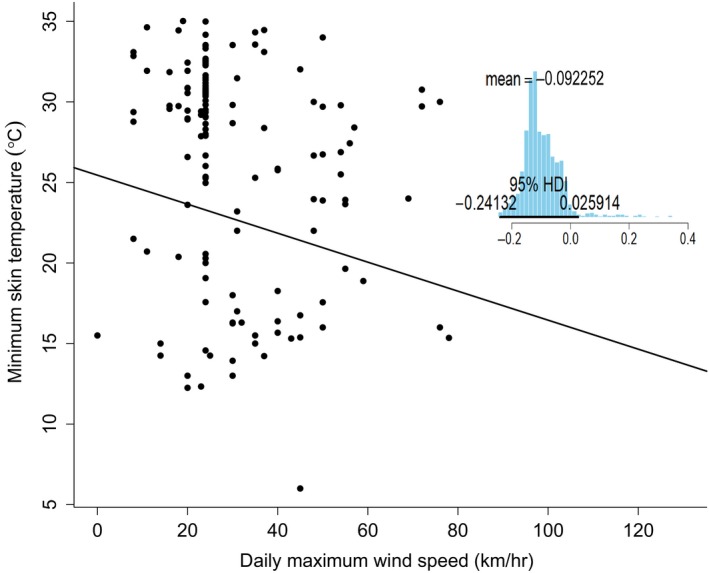
The effects of daily maximum wind speed on torpor depth in female *Myotis lucifugus* in Newfoundland from June to August 2016 and 2017. The black line is a linear regression based on the model‐averaged posterior estimates of the slope and intercept (*y* = −0.09*x* + 25.45). The posterior distribution of maximum wind speed for the highest ranked model is adjacent to the plot

Based on the model‐averaged posterior estimates, precipitation had a stronger effect on torpor frequency than maximum wind speed. An increase in maximum wind speed from 0 to 10 km/hr most likely increased torpor frequency by 1.35 times (exp[posterior estimate × unit of increase]) and an increase in precipitation from 0 to 10 mm most likely increased torpor frequency by 22.20 times. However, individuals started with a high probability of using torpor when maximum wind speed (*y*‐intercept = 0.86) and precipitation (*y*‐intercept = 0.89) were zero (Figure 4). Maximum wind speed was also an important variable for explaining variation in torpor depth (i.e., minimum *T*_sk_) and an increase in maximum wind speed from 0 to 10 km/hr most likely decreased minimum *T*_sk_ by 0.9°C (Figure 5). However, there was uncertainty in the estimate as the 95% HDI slightly overlapped zero and the shape of the distribution indicates a large amount of variation in posterior probabilities.

## 4 DISCUSSION

Our results indicate that combinations of intrinsic and weather variables explain torpor use in female temperate bats in cool, wet climates. This is consistent with previous studies (Dzal & Brigham, 2013; Johnson & Lacki, 2014; Rintoul & Brigham, 2014); however, we also quantified interindividual variation in torpor patterns. Interindividual variation in torpor use may have implications for fitness as differences in the timing of parturition and weaning may have different consequences for offspring survival (Frick, Reynolds, & Kunz, 2010; Kunz, Wrazen, & Burnett, 1998). Additionally, current methods of defining a torpor threshold may over‐or underestimate torpor use in some individuals (Barclay et al., 2001; McKechnie et al., 2007), which may influence the effect of reproductive condition and individual on some torpor patterns. Our study illustrates the importance of including individual effects within reproductive condition when explaining thermoregulatory strategies, which may be useful for understanding how individuals of populations will respond to environmental fluctuations or disease.

Our hypothesis that reproductive condition and interindividual variation explain torpor patterns was supported for torpor duration and depth, but not frequency. Based on the posterior estimates, we considered reproductive condition to have the greatest effect on torpor duration and the variable individual to have the greatest effect on depth. As predicted, and similar to other research (Dzal & Brigham, 2013; Johnson & Lacki, 2014; Lausen & Barclay, 2003), females in the pregnant and lactating conditions had shorter durations and shallower depths of torpor than the post‐lactating condition. Without the physiological costs to reproduction (Racey & Swift, 1981; Wilde et al., 1999), post‐lactating females may use long and deep torpor bouts to conserve energy in preparation for hibernation and reproduction the following spring (Johnson & Lacki, 2014; Jonasson & Willis, 2011; Kunz et al., 1998).

Supporting our prediction but contrary to other studies of *M. lucifugus* (Dzal & Brigham, 2013; Studier & O'Farrell, 1972), females in the not‐obviously‐pregnant and pregnant condition had longer durations and greater depths of torpor than the lactating condition. There were likely females in early pregnancy within the not‐obviously‐pregnant group in our study's sample and differences in torpor use between these two groups may be due to higher costs of torpor on fetal development during late pregnancy than early pregnancy (Racey et al., 1987; Racey & Swift, 1981). Contrary to our prediction and other studies of temperate bats (Audet & Fenton, 1988; Johnson & Lacki, 2014), females in the nonreproductive condition had shorter durations and shallower depths of torpor than the pregnant condition. This may be explained by differences in environmental conditions when each reproductive condition occurs and when females were sampled. Weather conditions at our study site were colder with greater wind speeds and lower insect abundance (Fouts, 2018) when pregnant and not‐obviously‐pregnant females were sampled, resulting in higher energetic demands than when lactating and nonreproductive females were sampled. Females may maximize reproductive success in these environments by delaying parturition until weather conditions are more favorable for juvenile growth (Gillooly, Charnov, West, Savage, & Brown, 2002; Willis et al., 2006) and insect availability is higher (Anthony et al., 1981). At our study site, the earliest approximated parturition date was 2–6 weeks later than that documented for other populations of *M. lucifugus* (Dzal & Brigham, 2013; Frick et al., 2010; Kunz, 1971). In response to the late parturition dates, lactating females may reduce their torpor use compared to pregnancy to wean young as early as possible (Frick et al., 2010; Kunz et al., 1998). Based on capture dates, we speculate that the juvenile development period in our study was approximately two weeks, which is shorter than studies at lower latitudes (e.g., Iowa), where volant juveniles were found 4–5 weeks after parturition (Kunz, 1971). Therefore, female individuals in different geographic regions may use different thermoregulatory strategies to maximize reproductive success.

Similar to other studies on mammalian torpor use (Canale, Perret, Thery, & Henry, 2011; Dammhahn et al., 2016; Vuarin et al., 2013), there was interindividual variation in torpor use within reproductive condition and under similar environmental conditions. The individuals with the most negative and most positive posterior estimates for torpor duration were pregnant, suggesting more interindividual variation during pregnancy, although the sample sizes differed among reproductive conditions. Potential explanations for interindividual variation in torpor use include genetic variation in torpor‐related traits and body condition (Lane et al., 2011; Rambaldini & Brigham, 2008; Vuarin et al., 2013). Individuals in better body condition have been found to limit torpor use and the associated negative consequences due to sufficient fat reserves for maintaining normothermy (Rambaldini & Brigham, 2008; Stawski & Geiser, 2010). However, individuals in poor body condition have also been found to limit torpor use due to insufficient fat reserves for arousal or to optimize foraging (Vuarin et al., 2013). Further research on the causes of interindividual variation will be useful for identifying the fitness implications of various thermoregulatory responses (Dammhahn et al., 2016; Lane et al., 2011) and determining how individuals of populations will respond to environmental fluctuations (Canale et al., 2011; Doucette, Brigham, Pavey, & Geiser, 2011; Vuarin et al., 2013).

Our hypothesis that weather conditions explain torpor patterns was supported for torpor frequency and depth. As predicted, high wind speed and precipitation were associated with increased torpor frequency and high wind speeds resulted in greater depths of torpor. Given the effect of wind and precipitation on heat loss during flight (Klug‐Baerwald et al., 2016; Voigt et al., 2011) and on insect activity (Anthony et al., 1981; Racey et al., 1987), bats may forego foraging and increase their frequency of torpor use (Klug & Barclay, 2013). Despite wind and precipitation being the most important variables for explaining torpor frequency, individuals started with a high probability of using torpor when wind and precipitation were at zero. This suggests that other variables may affect torpor frequency, including previous weather conditions (Klug & Barclay, 2013), time spent foraging (Rintoul & Brigham, 2014), and insect availability (Anthony et al., 1981; Racey et al., 1987). Similarly, the varied effect of wind on torpor depth suggests a confounding influence from other unidentified variables. Our results demonstrate the importance of using a multivariate approach when evaluating the effects of environmental conditions on thermoregulatory patterns.

While the torpor threshold generally provided a reliable estimate of torpor frequency and duration for most individuals, it likely underestimated torpor use for some post‐lactating and not‐obviously‐pregnant individuals. Post‐lactating and not‐obviously‐pregnant females had *T*_sk_ patterns that involved maintaining low *T*_sk_ over one day, in which normothermia was never reached before emergence, and over multiple days. This produced torpor thresholds from 22 to 29°C, which resulted in measurements of torpor durations that were much shorter. Similar to other studies (Willis et al., 2006), our results suggest that females can use torpor for extended periods post‐hibernation, in which current methods used to define torpor based on *T*_sk_ (Barclay et al., 2001; McKechnie et al., 2007) are not always suitable for measuring torpor patterns during reproduction. Establishing concurrent measures of metabolic rates or *T*_b_ with *T*_sk_ to extrapolate a threshold (Willis, 2007) may better quantify the effect of intrinsic and weather variables on torpor patterns.

Our study demonstrates that individual female *M. lucifugus* may employ different thermoregulatory responses depending on intrinsic and weather variables, with torpor patterns varying among individuals within the same reproductive condition. Females in early spring (i.e., the not‐obviously‐pregnant condition) used torpor to the greatest extent which may be necessary for the survival and reproduction of this species in regions with cool spring weather (Willis et al., 2006). Climate change and disease may affect these thermoregulatory strategies, which may have implications for the population growth and persistence of small, insectivorous species (Francl, Ford, Sparks, & Brack, 2012; Frick et al., 2010; Rodenhouse, Christenson, Parry, & Green, 2009). Individuals with a higher degree of phenotypic flexibility may be more successful at persisting through environmental variations, such as those caused by climate change (Canale et al., 2011; Vuarin et al., 2013), or diseases affecting thermoregulation, such as white‐nose syndrome (Jonasson & Willis, 2011). Years with extreme weather conditions may influence insect abundance (Rodenhouse et al., 2009) and has been associated with lower reproductive rates and later reproductive timing in temperate bat species (Burles, Brigham, Ring, & Reimchen, 2009; Frick et al., 2010; Grindal, Collard, Brigham, & Barcl, 1992; Lewis, 1993). This is likely due to increased thermoregulatory costs and a reduction in food availability, which may lead females to increase their use of torpor or abandon reproduction due to energy shortages (Burles et al., 2009; Frick et al., 2010; Grindal et al., 1992). White‐nose syndrome is a fungal disease that has resulted in the deaths of over one million *M. lucifugus* in North America from 2005 to 2011 (Dzal, McGuire, Veselka, & Fenton, 2011). It disrupts torpor patterns during hibernation, causing bats to deplete fat stores more rapidly than they normally would (Blehert et al., 2009; Reeder et al., 2012). White‐nose syndrome may prevent deep or prolonged torpor use in pregnant females during cold spring weather, which may alter reproductive timing (Francl et al., 2012; Jonasson & Willis, 2011). A reduction in torpor use during spring may result in earlier parturition dates (Francl et al., 2012) that occur before warm *T*_a_ and higher insect availability, which may not be conducive for neonatal growth and survival (Gillooly et al., 2002; Willis et al., 2006). Future research on the causes of interindividual variation in torpor use and the fitness consequences of those thermoregulatory responses may help predict the effects of climate change and disease on populations of heterothermic species.

## CONFLICT OF INTEREST

None declared.

## AUTHOR CONTRIBUTIONS

N.K.B. and H.G.B. conceived this study. N.K.B. collected and analyzed data and led the writing of the manuscript. N.K.B. and H.G.B. contributed critically to drafts and approved the final manuscript.

## Data Availability

Data and code are available from the Dryad Digital Repository: https://doi.org/10.5061/dryad.c04tj85, https://doi.org/10.5061/dryad.c04tj85.
